# Association Between Use of Any of the Drugs Prescribed in Norway and the Subsequent Risk of Parkinson Disease: A Drug-wide Association Study

**DOI:** 10.1212/WNL.0000000000207899

**Published:** 2023-11-21

**Authors:** Julia Romanowska, Kjetil Bjornevik, Marianna Cortese, Julia A. Tuominen, Magne Solheim, Asieh Abolpour Mofrad, Jannicke Igland, Clemens R. Scherzer, Trond Riise

**Affiliations:** From the Department of Global Public Health and Primary Care (J.R., K.B., M.C., J.A.T., M.S., A.A.M., J.I., T.R.), University of Bergen, Norway; Department of Nutrition (K.B., M.C.), and Department of Epidemiology (K.B.), Harvard T.H. Chan School of Public Health; and Precision Neurology Program (C.R.S., T.R.), and APDA Center for Advanced Parkinson Research (C.R.S.), Harvard Medical School, Brigham and Women's Hospital, Boston, MA.

## Abstract

**Background and Objectives:**

The incidence rate of Parkinson disease (PD) has been increasing rapidly during the past years. Yet, no treatments exist to prevent or slow the progression of the disease. Moreover, we are unable to detect early disease stages during which intervention with disease-modifying therapies is most likely to succeed. The objective of this study was to perform an agnostic drug-wide association study estimating the association between the use of any of the drugs prescribed in Norway and the subsequent risk of PD.

**Methods:**

This registry-based cohort study use data from the entire Norwegian population between 2004 and 2019 linked to the Norwegian Prescription Registry, with more than 600 million individual prescriptions. Drug classes were screened according to Anatomical Therapeutic Chemical codes at level 2, corresponding to therapeutic subgroups. We used Cox regression models to estimate hazard ratios (HRs) and 95% CIs for the associations between drug classes and PD risk. All *p* values were corrected for multiple testing using the false discovery rate. In addition, we conducted sensitivity analyses of exposure definition as well as time-lag and dose-response analyses.

**Results:**

The study population comprised 3,223,672 individuals, 15,849 of whom developed PD during the follow-up. We identified 31 drug classes that were statistically significantly associated with PD risk in Norway during the follow-up. Drugs acting on the renin-angiotensin system (HR 0.92, 95% CI 0.89–0.95), corticosteroids for systemic use (0.88, 95% CI 0.84–0.93), and vaccines (0.89, 95% CI 0.82–0.96) were associated with a decreased risk of PD even up to 10 years before PD onset. Drug classes used to treat symptoms related to prodromal signs of PD, such as constipation, urological issues, and depression, were associated with an increased risk of subsequent diagnosis of PD with HRs of 1.6 (95% CI 1.49–1.73), 1.48 (1.42–1.53), and 1.94 (1.87–2.01), respectively.

**Discussion:**

This drug-wide study identified 31 drug classes that were associated with the PD risk change. It reveals the links of renin-angiotensin system medications, vaccines, and corticosteroids with PD risk and suggests that monitoring drug usage using pharmacoepidemiology may allow identifying individuals with prodromal PD.

## Introduction

Parkinson disease (PD) is a brain disorder for which the incidence is growing fast and is expected to markedly increase by 2040.^1^ Yet, the details of the pathogenesis are unclear, and there are no available treatments that can prevent or slow the disease progression. The median cost of developing a new therapeutic drug from scratch has been estimated at $1,141.7 million (95% CI, $888.1 million–$1,480.8 million)^2^ and includes years of screening, chemistry, and preclinical testing until any compound can reach human studies. When a drug does finally enter trials, 67%–96% of molecules fail,^2,3^ in part due to adverse effects and the limited ability of animal models to predict efficacy in humans.^4^ Repurposing existing drugs represents an attractive alternative to traditional drug development.^5^

We have conducted hypothesis-free analyses using data from Norwegian health registries to evaluate associations between any of the drugs used in Norway over a period of 15 years and the risk of developing PD. In a previous study, we linked drug effects demonstrated through experiments in cells and animals to corresponding associations in humans using these registries.^6^

In the present analysis, we used a systematic, drug-wide, agnostic approach aiming to identify drugs that (1) could be repurposed to treat PD (drugs associated with reduced risk of PD) or (2) could help explain PD pathogenesis (drugs associated with increased risk of PD). Although the results presented here cannot give a direct causal relationship, we believe that this hypothesis-free method is valuable as a first step in the drug repurposing pipeline. Such drug-wide screening approach might result in new avenues for further research that would have been otherwise overlooked when a clear hypothesis is present. A recent review shows that novel approaches are crucial in finding possible treatment options for PD.^7^ This is a study of the Drug Repurposing for Neurological Diseases Project (DRONE),^8^ aiming at establishing a novel framework for developing new treatment options for PD, multiple sclerosis, Alzheimer disease, and amyotrophic lateral sclerosis. Here, we present a hypothesis-free screening of all the drugs available on the Norwegian market for associations with subsequent risk of PD.

## Methods

### Study Population and Design

Our registry-based cohort study included all individuals registered as living in Norway on January 1, 2004 (n = 4.6 million individuals). We obtained demographic data, including sex, year of birth, year of death, and yearly emigration status, and educational level from Statistics Norway (SSB). Individuals were linked to the Norwegian Prescription Database (NorPD) to obtain information on all prescribed drugs in Norway from 2004 to 2019 and linked to the nationwide Norwegian Patient Registry (NPR) to obtain all discharge diagnoses of PD from 2009 to 2019 (*ICD-10* code G20). The linkage between registries is unambiguous thanks to the Norwegian personal identification number that is assigned to every Norwegian citizen.

From NorPD, we had information on the month and year of collection of each prescription, the Anatomical Therapeutic Chemical (ATC) code of the drug, and details about the amount of drug prescribed in each prescription, as well as reimbursement codes from specialists and general practitioners. The ATC code comprised 5 levels of grouping the drugs according to where they act in the body and what therapeutic or molecular features they share. Here, we present the results for groups of drugs defined by the second level of the ATC code, which indicates therapeutic subgroups (eTable 1, links.lww.com/WNL/D145). We chose this ATC level because we wanted to focus on trends in the population while minimizing the noise and correlations between the usage of drugs targeting similar symptoms.

**Figure 1:**
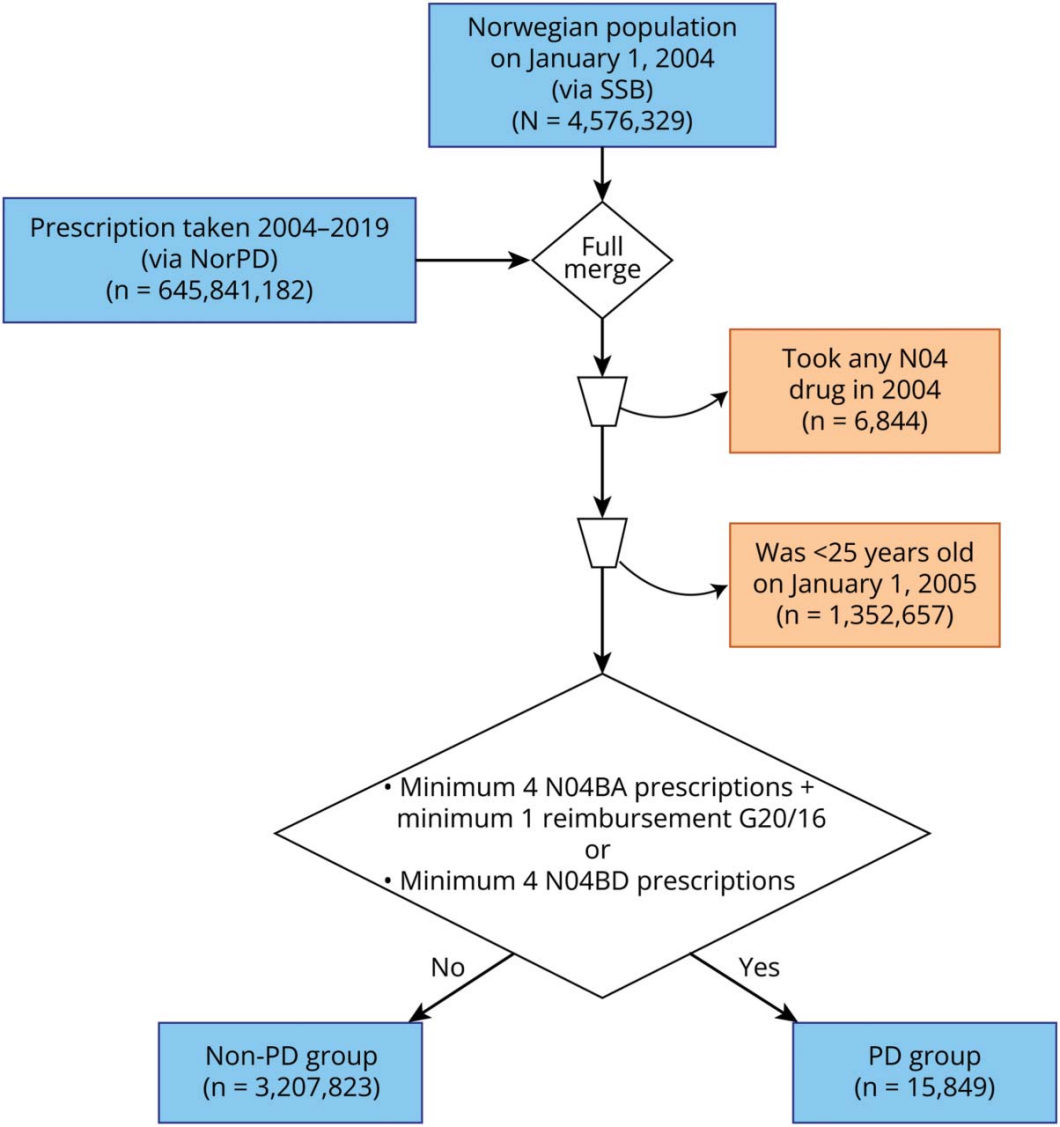
Study Population Overview NorPD = Norwegian Prescription Database; PD = Parkinson disease; SSB = Statistics Norway.

For the analyses, we excluded individuals who were younger than 25 years at the start of follow-up (January 1, 2005), as these had minimal risk of PD, a late-onset aging-dependent disease, during the follow-up. To include only incident cases of PD (i.e., patients who received a diagnosis during the follow-up period in our study), we excluded individuals who had at least 1 prescription of any anti-Parkinson drug (ATC group N04) in 2004 (Figure 1). This resulted in a study population of 3,223,672 individuals (51% female).

### Assessment of Exposure

Individuals who had at least 2 prescriptions of any drug from a given ATC group throughout the follow-up time were considered exposed to this drug group. Individuals were considered exposed from the time of the second prescription of the drug in question and during the rest of the study period. To minimize unreliable results, we restricted the analyses to the ATC groups for which there were at least 5 patients with PD who had taken these drugs. A total of 86 of 94 ATC groups fulfilled this criterion.

In addition, we ran sensitivity analyses where we defined individuals as exposed only after the time of having received at least 4 or 8 prescriptions of the relevant drug group.

### Assessment of Outcome

We defined patients with PD as individuals who either (1) had at least 4 prescriptions of levodopa (ATC group N04BA) and had any prescription (not necessarily for levodopa) with reimbursement code for PD (either *ICD-10*: G20 or a local code [PktNr: 16] used in the NorPD during 2004–2008) or (2) had at least 4 prescriptions of monoamine oxidase B (MAO-B) inhibitors (ATC group N04BD) (Figure 1). The time of onset was defined as the date of the first prescription of any anti-Parkinson drug (ATC group N04). The identified PD cases were checked against data on hospitalizations and outpatient visits from NPR. Among 9,696 identified cases with PD onset date during 2010–2019, 92% were registered at least once with G20 as the primary diagnosis in NPR. The mean time between the date of the first prescription of any N04 drug and the date of the first primary G20 diagnosis registered in the NPR was 34.3 days.

### Statistical Methods

For the original analyses, we used Cox proportional hazard models using the age of the individuals as the time scale to estimate hazard ratios (HRs) and 95% CIs for the association between the exposure to drug groups and subsequent risk of PD during follow-up. Exposure to each drug group was included as a time-dependent variable in separate models. All models were adjusted for sex and education level. The educational level was analyzed as a categorical variable with 5 levels: primary, secondary, tertiary short (up to 3 years), tertiary long (more than 3 years), and missing. In total, 1.8% of the individuals had missing information on education. Individuals were followed until PD onset, death, emigration, or end of the study (December 31, 2019), whichever occurred first. All analyses were repeated stratified on sex. All *p* values were corrected for multiple testing using the Benjamini and Yakutieli method to control the false discovery rate (FDR).

Moreover, we conducted time-lag and dose-response analyses. In the time-lag analyses, the end of follow-up was shifted to 5, 8, and 10 years before the PD index date or censoring date. This was done to evaluate the possibility of false-positive results because of reverse causality. In the dose-response analyses, we used the number of prescriptions within a drug group as a proxy for dose. The strata were created based on the following percentiles: 25th, 50th, 75th, and 90th, calculated among those with at least 2 prescriptions. The dose was then included in the Cox regression models as a time-varying covariate. The models were adjusted for age (as the time scale), sex, and education.

The analysis was performed in R (version 4.1.3), using the survival package.^9^ The figures were made using tidyverse^10^ and patchwork^11^ R packages. The tables were prepared with gt.^12^ The interactive visualization of the results was written in Rmarkdown, using flexdashboard,^13^ htmlwidgets,^14^ crosstalk,^15^ and plotly^16^ R-packages.

### Standard Protocol Approvals, Registrations, and Patient Consents

The project received ethical approval and exemption from informed consent from the Western Norway Regional Committee for Medical and Health Research Ethics (REK West: 2017/1508). Permissions to use and merge data were obtained from NorPD, NPR, and SSB.

### Data Availability

The data are available from the registries for researchers who apply to the Norwegian Regional Ethics Committee. The analysis code, the interactive visualizations, and results are available on GitHub at jromanowska.github.io/Norwegian_drug_screening_ATC_level2.

## Results

We identified a total of 15,849 individuals who were diagnosed with PD during the follow-up period from 2005 to 2019. The mean age at diagnosis was 70.5 years, and a total of 60% were male patients. The proportions of patients who had received levodopa medication and MAO-B inhibitors were 96.7% and 46.2%, respectively.

Of a total of 86 drug classes analyzed at level 2 of ATC, we found that 10 classes were associated with a *reduced* PD risk and 21 classes were associated with an *increased* PD risk after adjusting for FDR (eFigure 1, links.lww.com/WNL/D145).

**Figure 2:**
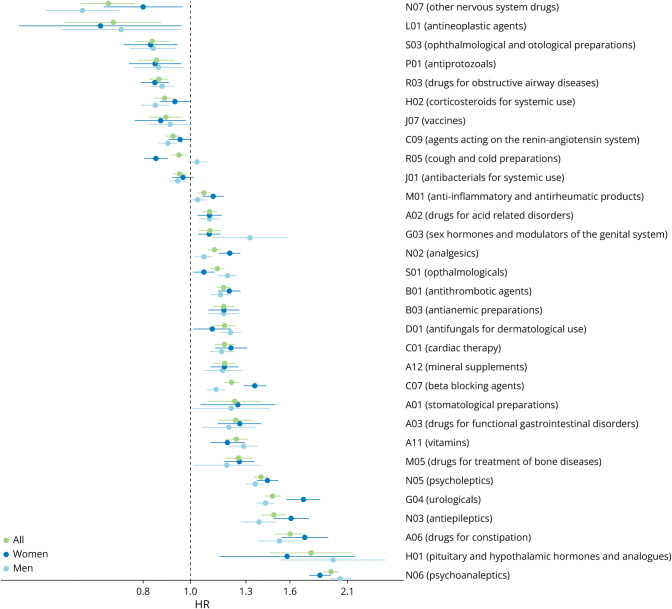
Comparing the Results From the Significant Drug Groups Between the Analyses Including Entire Population and the Sex-Stratified Analyses The points and lines show hazard risk (HR) estimates and CIs, respectively, from the time-varying Cox regressions.

Figure 2 shows all significant results of the original analyses on the entire data set and stratified by sex. Table 1 presents more details on the significant results of the analysis on the entire population, while the significant results of the sex-stratified analysis are presented in eTable 2 (links.lww.com/WNL/D145).

**Table 1:**
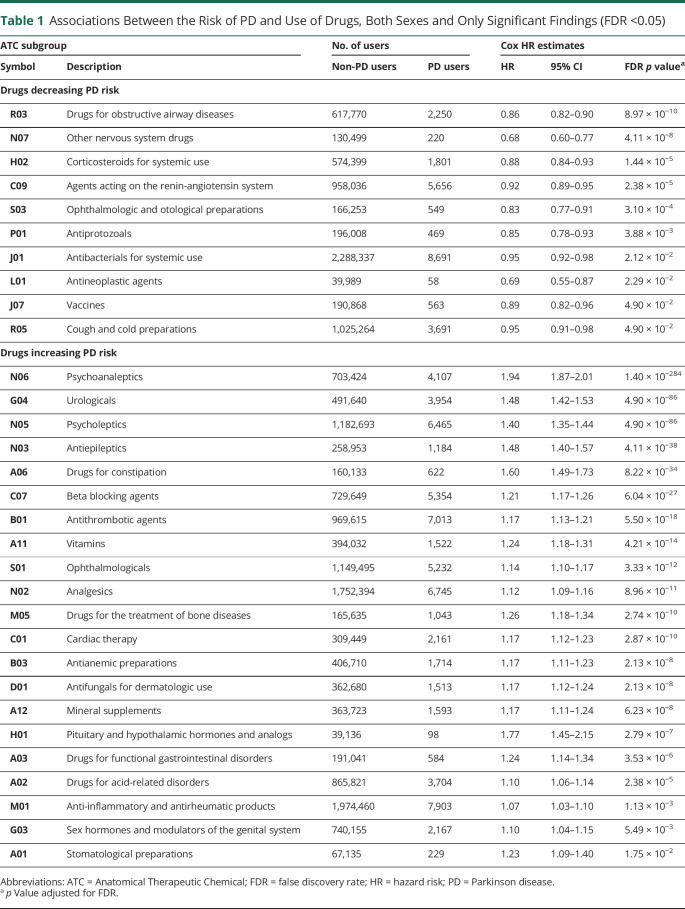
Associations Between the Risk of PD and Use of Drugs, Both Sexes and Only Significant Findings (FDR <0.05)

ATC subgroup		No. of users		Cox HR estimates		
Symbol	Description	Non-PD users	PD users	HR	95% CI	FDR *p* value^a^
Drugs decreasing PD risk						
R03	Drugs for obstructive airway diseases	617,770	2,250	0.86	0.82–0.90	8.97 × 10^−10^
N07	Other nervous system drugs	130,499	220	0.68	0.60–0.77	4.11 × 10^−8^
H02	Corticosteroids for systemic use	574,399	1,801	0.88	0.84–0.93	1.44 × 10^−5^
C09	Agents acting on the renin-angiotensin system	958,036	5,656	0.92	0.89–0.95	2.38 × 10^−5^
S03	Ophthalmologic and otological preparations	166,253	549	0.83	0.77–0.91	3.10 × 10^−4^
P01	Antiprotozoals	196,008	469	0.85	0.78–0.93	3.88 × 10^−3^
J01	Antibacterials for systemic use	2,288,337	8,691	0.95	0.92–0.98	2.12 × 10^−2^
L01	Antineoplastic agents	39,989	58	0.69	0.55–0.87	2.29 × 10^−2^
J07	Vaccines	190,868	563	0.89	0.82–0.96	4.90 × 10^−2^
R05	Cough and cold preparations	1,025,264	3,691	0.95	0.91–0.98	4.90 × 10^−2^
Drugs increasing PD risk						
N06	Psychoanaleptics	703,424	4,107	1.94	1.87–2.01	1.40 × 10^−284^
G04	Urologicals	491,640	3,954	1.48	1.42–1.53	4.90 × 10^−86^
N05	Psycholeptics	1,182,693	6,465	1.40	1.35–1.44	4.90 × 10^−86^
N03	Antiepileptics	258,953	1,184	1.48	1.40–1.57	4.11 × 10^−38^
A06	Drugs for constipation	160,133	622	1.60	1.49–1.73	8.22 × 10^−34^
C07	Beta blocking agents	729,649	5,354	1.21	1.17–1.26	6.04 × 10^−27^
B01	Antithrombotic agents	969,615	7,013	1.17	1.13–1.21	5.50 × 10^−18^
A11	Vitamins	394,032	1,522	1.24	1.18–1.31	4.21 × 10^−14^
S01	Ophthalmologicals	1,149,495	5,232	1.14	1.10–1.17	3.33 × 10^−12^
N02	Analgesics	1,752,394	6,745	1.12	1.09–1.16	8.96 × 10^−11^
M05	Drugs for the treatment of bone diseases	165,635	1,043	1.26	1.18–1.34	2.74 × 10^−10^
C01	Cardiac therapy	309,449	2,161	1.17	1.12–1.23	2.87 × 10^−10^
B03	Antianemic preparations	406,710	1,714	1.17	1.11–1.23	2.13 × 10^−8^
D01	Antifungals for dermatologic use	362,680	1,513	1.17	1.12–1.24	2.13 × 10^−8^
A12	Mineral supplements	363,723	1,593	1.17	1.11–1.24	6.23 × 10^−8^
H01	Pituitary and hypothalamic hormones and analogs	39,136	98	1.77	1.45–2.15	2.79 × 10^−7^
A03	Drugs for functional gastrointestinal disorders	191,041	584	1.24	1.14–1.34	3.53 × 10^−6^
A02	Drugs for acid-related disorders	865,821	3,704	1.10	1.06–1.14	2.38 × 10^−5^
M01	Anti-inflammatory and antirheumatic products	1,974,460	7,903	1.07	1.03–1.10	1.13 × 10^−3^
G03	Sex hormones and modulators of the genital system	740,155	2,167	1.10	1.04–1.15	5.49 × 10^−3^
A01	Stomatological preparations	67,135	229	1.23	1.09–1.40	1.75 × 10^−2^

Abbreviations: ATC = Anatomical Therapeutic Chemical; FDR = false discovery rate; HR = hazard risk; PD = Parkinson disease.

a *p* Value adjusted for FDR.

### Drugs Associated With Reduced PD Risk

We identified an association between drugs acting on the renin-angiotensin system (RAS) (ATC class C09) and decreased the risk of PD (HR 0.92; 95% CI 0.89–0.95). By contrast, beta-blockers (C07) and drugs of cardiac therapy (C01, including digitalis, antiarrhythmics) were linked to increased PD risk (C07: HR 1.21; 95% CI 1.17–1.26 and C01: HR 1.17; 95% CI 1.12–1.23). We found no significant association with lipid-modifying agents (C10: HR 1.00) or calcium channel blockers (C08: HR 0.98).

Of the 10 classes related to a lower risk of PD, there were 2 classes associated with a reduced PD risk of 30% or more, which were other nervous system drugs (N07) and antineoplastic drugs (L01). Moreover, we found associations between reduced PD risk and the use of vaccines (J07), antibacterials for systemic use (J01), and antiprotozoals (P01), as well as corticosteroids for systemic use (H02). The association with diabetes drugs was not significant after controlling for multiple testing (A10: HR 0.93, 95% CI 0.87–0.99).

### Subgroup Analysis of the RAS Drugs

Because previous studies have shown an effect of the angiotensin receptor blockers (ARBs), a subgroup of the RAS drugs (C09), but not for the angiotensin-converting enzyme inhibitors (ACEIs) subgroup, we estimated the effect of these groups separately. We found a reduced risk for both subgroups, HR 0.93 (95% CI 0.89–0.96) for ARBs and HR 0.92 (95% CI 0.88–0.96) for ACEIs.

### Drugs Associated With Increased PD Risk

Our screen revealed 21 drug classes associated with an increased risk of PD (Table 1). Many of these drug classes are used to treat symptoms commonly observed in individuals with prodromal PD. These include urologicals (G04: HR 1.48; 95% CI 1.42–1.53), drugs for constipation (A06: HR 1.60; 95% CI 1.49–1.73), and psychoanaleptics (N06: HR 1.94; 95% CI 1.87–2.01). The heterogeneous class of psycholeptic drugs (N05), which includes both antipsychotics and benzodiazepines, was associated with increased PD risk (HR 1.40; 95% CI 1.35–1.44). In addition, several drugs used in the treatment of bone diseases (M05), antiepileptics (N03), and pituitary and hypothalamic hormones and analogs (H01) were associated with markedly increased PD risk.

### Differences in Drug Use–Associated PD Risk Between Men and Women

In analyses stratified by sex, we found that the reduced PD risk associated with other nervous system drugs (N07) was stronger in men (HR 0.60) than in women (HR 0.80, *p* value for interaction = 0.028) (Figure 2 and eTable 2, links.lww.com/WNL/D145). Interestingly, cough and cold preparations (R05) were associated with a lower PD risk only in women (HR 0.85 vs HR 1.03 in men, *p* value for interaction = 9·× 10^−7^). Furthermore, the overall increased PD risk for urologicals (G04) was significantly stronger among women (HR 1.71) compared with men (HR 1.43; *p* value for interaction = 8 × 10^−6^).

### Sensitivity Analyses: Varying Exposure Definition

When the definition of being exposed to a drug group was changed from minimum 2 prescriptions to minimum 4 or 8, the resulting HR estimates did not change markedly (eFigure 2, links.lww.com/WNL/D145). Most effect estimates stayed within the CI limits of the estimates from the original analysis and the CIs became wider. The association between the use of drugs for functional gastrointestinal disorders (A03) and increased PD risk became stronger in the sensitivity analyses.

### Investigating Time Dependence of the Exposure Window

The selected results from the time-lag analyses with lags of 5, 8, and 10 years are presented in eFigure 3 (links.lww.com/WNL/D145), and all the results are available online in the GitHub repository. The drug groups that were still statistically significantly associated with an increased PD risk after anticipating the end of follow-up date by 10 years were urologicals (G04) and psychoanaleptics (N06), whereas drug groups still significantly associated with a decreased PD risk were corticosteroids for systemic use (H02), antibacterials for systemic use (J01), drugs for obstructive airway diseases (R03), ophthalmologic and otological preparations (S03), and other nervous system drugs (N07). The drugs acting on the renin-angiotensin system were still significantly reducing the risk of PD up to 8 years before the diagnosis.

### Dose-Response Analyses

eFigure 4 (links.lww.com/WNL/D145) shows the selected results of the dose-response analyses for the drug groups that were significantly associated with PD risk in the original analyses, while all the results are available online in the GitHub repository. There was a trend in HR estimates for several ATC groups that where significantly associated with a decreased risk of PD: RAS drugs (C09), corticosteroids for systemic use (H02), antibacterials for systemic use (J01), and drugs for obstructive airway diseases (R03).

## Discussion

In this long-term nationwide study, we identified 31 therapeutic drug classes that were associated with the risk of later being diagnosed with PD. We found marked associations with a reduced risk of PD, including drugs acting on the renin-angiotensin system (C09), vaccines (J07), antibiotics (J01), and corticosteroids for systemic use (H02).

The association with a reduced PD risk for drugs acting on RAS (C09) has previously been described among others in an early review of experimental data and clinical observations,^17^ 2 retrospective cohort studies of the risk for PD among 107,207 patients treated for hypertension^18^ and among 62,228 patients with ischemic heart disease,^19^ and 1 nationwide screening using a nested case-control study of 10,183 PD cases and 67,849 controls.^20^ Furthermore, a clinical prospective study of 107 patients with PD found that treatment with ARB improved cognitive function.^21^ The 3 epidemiologic studies found that the use of the ARBs was associated with a reduced PD risk, whereas no association was found for the ACEIs. Here, we found a reduced PD risk for users of both subgroups of the RAS drugs, compatible with a role for the RAS system itself rather than the specific drugs. Another explanation for the association with RAS drugs could be related to orthostatic hypotension, a prodromal sign of PD, which reduces the likelihood of being prescribed antihypertensive medications. However, the fact that the association persisted after moving the exposure window up to 8 years before PD diagnosis and that we found no association with another large group of antihypertensives, the calcium channel blockers (C08), argues against this. Analogously, the association between another cardiovascular drug class, beta blocking agents (C07), and increased risk of PD might be due to these drugs being prescribed for undifferentiated tremor, that is, another prodromal sign of PD. This interpretation is supported by the time-lag analysis showing that the association disappeared when introducing a lag of 5 years. These findings are also not compatible with this association being related to more general risk factors for hypertension that have been found to be related to a reduced PD risk, such as smoking, but instead suggest a role of specific mechanisms related to the RAS.

The findings for vaccines (J07), antibacterials (J01), and corticosteroids for systemic use (H02) might be compatible with the existence of pathologic pathways related to inflammation and immune response, some already known^22,23^ and new ones. In our results, both H02 and J01 retained their significant association with reduced PD risk up to 10 years before diagnosis, and both drug classes showed a dose-dependent trend suggesting a stronger effect for higher doses. On the other hand, this is in contrast to the well-known “Parkinson personality” where individuals follow medical instructions and thus might seek vaccination more often. However, the decreased risk being associated with not only 1 but 3 different drug groups gives us more confidence in the finding. Moreover, newly published results indicate increased PD risk 10 years or more after influenza and other infections in the Danish population.^24^ Similarly, a study on the Swedish population found an increased risk of PD more than 5 years after a hospital-treated infection.^25^ Another work showed associations between various virus exposures and neurodegenerative diseases, using Finnish registries as discovery cohort and replicating these results in UK Biobank.^26^ Moreover, a recent study of a cohort of older adults showed a reduced risk of Alzheimer disease after influenza vaccination.^27^

A recent review of the role of lipid-modifying agents showed that the use of these drugs was associated with a moderately decreased PD risk, but the authors concluded that a study with a large sample size and longer follow-up was needed to either confirm or refute the findings.^28^ In the present large nationwide study including more than 15,000 PD cases with up to 15 years of follow-up, we could not confirm the association with statins. Moreover, recently published results of a randomized clinical trial of simvastatin (C10AA01) demonstrated no effect on moderate PD.^29^

The long prodromal phase of PD may precede motor symptoms by more than a decade.^30^ These include constipation,^31^ depression,^31^ and possibly bladder disturbances.^32^ We found an increased PD risk associated with the use of several drug classes related to these conditions, including antidepressive medications (represented in the class psychoanaleptics; N06), drugs for constipation (A06), drugs for functional gastrointestinal disorders (A03), and urologicals (G04). The association with an increased PD risk for urologicals was interestingly stronger among women compared with men. All mentioned drug groups, apart from A06, retained the statistically significant association with increased PD risk even up to 8 or 10 years before the onset of the disease. Moreover, there was a trend of increasing risk with dose for drugs for functional gastrointestinal disorders, whereas the other drug groups showed a trend toward no association as the dose increased.

Another ATC class acting on the nervous system, psycholeptics (N05), was also associated with higher PD risk in our data. One subclass of psycholeptics, antipsychotics (N05A), can cause parkinsonism as an unwanted adverse event by blocking dopaminergic transmission.^33,34^ This category also includes benzodiazepines, which could be linked to some prodromal signs, including sleep disorders or anxiety. Furthermore, our time-lagged results showed a steep decrease in the HR estimates already 5 years before PD diagnosis.

We found an association between antiepileptics (N03) and increased risk of PD, which was also recently reported in a UK Biobank substudy.^35^ The antiepileptic valproic acid is linked to drug-induced tremors,^36^ which might lead to either misdiagnosis of PD or an earlier diagnosis of true PD, because of better health monitoring. Moreover, the HR in our time-lagged results decreased quickly toward 1 (no effect) already 5 years before PD diagnosis, arguing for a reverse causal explanation of this association.

The association between the use of pituitary and hypothalamic hormones and analogs (H01) and higher PD risk is unclear. The HR did not change significantly in the time-lagged analyses, although the CIs became wider. Drugs from this group were used mainly against malignant tumors, malfunction of pituitary gland, and acromegaly among the controls while mainly against enuresis, malignant tumors, and malfunction of pituitary gland among the PD cases (data not shown). This association might also be related to yet unknown disease mechanisms.

These multiple associations between drug use and increased PD risk suggest that it may be possible to identify individuals with prodromal PD at scale by using electronic health data searches as prodromal biomarkers. High-throughput, low-cost screening tools for prodromal PD are critically needed and currently not available. The lack of such prodromal screens is a key obstacle for conducting preventive drug trials in PD which require a large numbers of prodromal participants. For disease causality, in these cases, the prodromal disease syndrome is believed to be the cause for a patient to take such medications (so-called “reverse causation” or confounding by preclinical disease activity); the medication is not hypothesized to be etiologically related to the disease pathobiology. When the disease pathology over time progresses from the prodromal to the classical motor symptoms, a clinical diagnosis of PD is established.

Reverse causality is less likely for the drug classes that were associated with a *reduced* risk of PD, as treatment indications (conditions or symptoms) for these drugs would have to occur less frequently among individuals with prodromal PD compared with the general population to explain the associations.

Still, the indication for some of these drug groups could be related to known risk factors, such as smoking, which consistently has been associated with a lower risk of PD,^37^ although the underlying pathobiological mechanism is unknown and clinical trials with nicotine failed.^38^ Drugs used for nicotine dependency represent the largest group within the N07 class (other nervous system drugs) probably explaining the association with reduced PD risk for this class.

The drugs for obstructive airway diseases (R03, including β2-agonists) were shown to be related to reduced PD risk in newly published studies on French national health data^39^ and Finnish health data.^20^ Specifically, β2-agonists have been previously investigated for a role in PD. Mechanistically, selective β2AR activation suppresses the degeneration of nigral dopamine neurons, prevents loss of striatal dopamine, and rescues movements in multiple rodent models of PD.^6,40,41^ Molecularly, β2 agonists reduce α-synuclein expression^6,42^ and microglial activation.^41^ Epidemiologically, the significant association between β2-agonists and the risk of PD was confirmed in a recent meta-analysis.^43^ However, there remains considerable controversy regarding the interpretation of the epidemiologic association (for discussion, see References 44-References 46). Notably, the R03 drug class includes also other treatments for chronic obstructive lung disease where smoking is a major risk factor, and this study design does not allow to shed light on the role of this possible confounding. For example, Hopfner et al.^44^ found inconsistent dose-response and exposure-time relationships between β2 agonists and risk, and a recent study^47^ did not support an association. By contrast, the only study directly adjusting for smoking (not just chronic obstructive pulmonary disease [COPD]) found a significant association between β2 agonists and reduced PD risk,^48^ and a follow-up study from Norway reports that after the exclusion of patients with COPD, corticosteroids and anticholinergics were no longer inversely associated with PD risk, whereas β2AR agonists remained associated.^46^

Furthermore, the findings on antibiotics (J01) could also partly be related to smoking as smokers more often experience upper throat infections.^49^ Similarly, the lower previous use of antineoplastic drugs (L01) among PD cases might also be related to a lower risk of smoking-related cancers in this group.^50^ On the other hand, the time-lagged results for 3 of the mentioned drug groups (N07, J01, and R03) showed that the associations with decreased PD risk stayed significant even up to 10 years before the diagnosis.

Our study has several strengths. We analyzed complete data from a large population aged 25 years and older, identifying almost 16,000 new PD cases during a follow-up of 15 years. Furthermore, more than 600 million individual prescriptions were evaluated for possible associations with PD risk giving us substantial statistical power. Finally, we conducted several additional analyses to evaluate changes in estimates introduced by varying dose or time lags for the exposure. Our study also has some limitations. The diagnosis of PD was based on the prescriptions of anti-Parkinson drugs, which might have caused a reduced sensitivity or specificity. Still, for the period where data from the NPR were available (2010–2019), as many as 92% were diagnostically confirmed. Moreover, we considered a person to be exposed to a drug group after collecting minimum 2 prescriptions within the exposure time window. However, our sensitivity analyses did not show major changes in results when increasing the minimum number of prescriptions from 2 to 4 or 8. Furthermore, as is inherent to any observational study, the results may be affected by unmeasured confounding.

Our approach of analyzing drug groups as opposed to single drugs has both advantages and disadvantages. On the disadvantage side, we are not able to pinpoint a specific drug that might be a good candidate for repurposing and some associations might be due to the underlying condition, not the drug usage. This warrants new analyses and experiments, which we are now exploring. However, grouping the drugs according to their therapeutic subgroup minimizes correlations between drugs given for the same disease and minimizes the multiple testing burden.

In conclusion, this drug-wide long-term study identified 31 drug classes associated with the risk of PD. It confirmed the link of renin-angiotensin system medications with PD and strengthened the evidence of inflammation as a possible underlying mechanism for the development of PD. Our results suggest that pharmacoepidemiologic surveillance may allow identifying previously elusive individuals with prodromal PD.

## Appendix Authors

**Table:**

Name	Location	Contribution
Julia Romanowska, PhD	Department of Global Public Health and Primary Care, University of Bergen, Norway	Study concept or design; analysis or interpretation of data
Kjetil Bjornevik, MD, PhD	Department of Global Public Health and Primary Care, University of Bergen, Norway; Department of Nutrition, and Department of Epidemiology, Harvard T.H. Chan School of Public Health, Boston, MA	Study concept or design
Marianna Cortese, MD, PhD	Department of Global Public Health and Primary Care, University of Bergen, Norway; Department of Nutrition, Harvard T.H. Chan School of Public Health, Boston, MA	Study concept or design
Julia A. Tuominen, MPsych	Department of Global Public Health and Primary Care, University of Bergen, Norway	Analysis or interpretation of data
Magne Solheim, MSc	Department of Global Public Health and Primary Care, University of Bergen, Norway	Analysis or interpretation of data
Asieh Abolpour Mofrad, PhD	Department of Global Public Health and Primary Care, University of Bergen, Norway	Analysis or interpretation of data
Jannicke Igland, PhD	Department of Global Public Health and Primary Care, University of Bergen, Norway	Major role in the acquisition of data; study concept or design; analysis or interpretation of data
Clemens R. Scherzer, MD	Precision Neurology Program, and APDA Center for Advanced Parkinson Research, Harvard Medical School, Brigham and Women's Hospital, Boston, MA	Major role in the acquisition of data; study concept or design
Trond Riise, PhD	Department of Global Public Health and Primary Care, University of Bergen, Norway; Neuro-SysMed, Haukeland University Hospital, Bergen, Norway; Precision Neurology Program, Harvard Medical School, Brigham and Women's Hospital, Boston, MA	Major role in the acquisition of data; study concept or design

## Glossary

ACEI: angiotensin-converting enzyme inhibitor
ARB: angiotensin receptor blocker
ATC: Anatomical Therapeutic Chemical
COPD: chronic obstructive pulmonary disease
FDR: false discovery rate
HR: hazard ratio
*ICD-10*: *International Classification of Diseases, 10th Revision*
MAO-B: monoamine oxidase B
NorPD: Norwegian Prescription Database
NPR: Norwegian Patient Registry
PD: Parkinson disease
RAS: renin-angiotensin system
SSB: Statistics Norway
